# Different treatment strategies versus a common standard arm (CSA) in patients with newly diagnosed AML over the age of 60 years: a randomized German inter-group study

**DOI:** 10.1007/s00277-023-05087-8

**Published:** 2023-01-25

**Authors:** Dietger Niederwieser, Thomas Lang, Rainer Krahl, Thomas Heinicke, Georg Maschmeyer, Haifa Kathrin Al-Ali, Sebastian Schwind, Madlen Jentzsch, Michael Cross, Christoph Kahl, Hans-Heinrich Wolf, Herbert Sayer, Antje Schulze, Peter Dreger, Ute Hegenbart, Alwin Krämer, Christian Junghanss, Lars-Olof Mügge, Detlev Hähling, Carsten Hirt, Christian Späth, Norma Peter, Bernhard Opitz, Axel Florschütz, Kolja Reifenrath, Niklas Zojer, Sebastian Scholl, Wolfram Pönisch, Simone Heyn, Vladan Vucinic, Andreas Hochhaus, Carlo Aul, Aristoteles Giagounidis, Leopold Balleisen, Bernd Oldenkott, Peter Staib, Michael Kiehl, Wolfgang Schütte, Ralph Naumann, Hartmut Eimermacher, Bernd Dörken, Cristina Sauerland, Eva Lengfelder, Wolfgang Hiddemann, Bernhard Wörmann, Carsten Müller-Tidow, Hubert Serve, Christoph Schliemann, Rüdiger Hehlmann, Wolfgang E. Berdel, Markus Pfirrmann, Utz Krug, Verena S. Hoffmann

**Affiliations:** 1 University Leipzig, 04106 Leipzig, Germany; 2 Lithuanian University of Health Sciences, Kaunas, Lithuania; 3 Aichi Medical University, Nagakute, Japan; 4 Present Address: Institut für Medizinische Informationsverarbeitung, Biometrie und Epidemiologie (IBE), Ludwig Maximilian Universität München, München, Germany; 5 Dept. Hematology and Oncology, Otto-Von-Guericke-University, Magdeburg, Germany; 6 Dept. Hematology, Oncology and Palliative Care, Klinikum Ernst Von Bergmann, Potsdam, Germany; 7 Department of Internal Medicine IV, Oncology/Hematology, Krukenberg Cancer-Center, University Hospital Halle (Saale), Halle, Germany; 8 Dept. Internal Medicine, Clinic III – Hematology, Oncology and Palliative Care, Rostock University Medical Center, Rostock, Germany; 9 Dept. Hematology, Oncology and Palliative Care, Klinikum Magdeburg gGmbH, Magdeburg, Germany; 10 Dept. Hematology and Oncology, University Hospital, Halle, Germany; 11 Medizinische Klinik (Hämatologie, Stammzelltransplantation, Onkologie), Helios Klinikum Erfurt, Erfurt, Germany; 12 Medical Department V, University Hospital, Heidelberg, Germany; 13 Clinical Cooperation Unit Molecular Hematology/Oncology, German Cancer Research Center (DKFZ) and Dept. of Internal Medicine V, University, Heidelberg, Germany; 14 Department of Medicine, Clinic III (Hematology, Oncology, Palliative Medicine), Rostock University Medical Center, Rostock, Germany; 15 Innere Medizin III (Hämatologie, Onkologie Und Palliativmedizin), Hospital Zwickau, Germany; 16 Dept. Hematology and Oncology, Klinikum Schwerin, Schwerin, Germany; 17 Innere Medizin C, Universitätsmedizin Greifswald, Sauerbruchstraße, 17475 Greifswald, Germany; 18 Medizinische Klinik, Carl-Thieme-Klinikum GmbH, Cottbus, Germany; 19 St. Elisabeth Und St, Barbara Hospital Halle (Saale), Halle, Germany; 20 Klinikum Dessau, Dessau, Germany; 21 Klinikum, Zittau, Germany; 22 1St Medical Department, Center for Oncology and Hematology & Palliative Care, Klinik Ottakring, Vienna, Austria; 23 Hematology/Oncology, Universitätsklinikum Jena, Jena, Germany; 24 Klinik Für Hämatologie Und Onkologie, St. Johannes Hospital, Duisburg, Germany; 25 Dept. Oncology, Hematology and Palliative Care, Marienhospital Düsseldorf, Düsseldorf, Germany; 26 Dept. Medicine II, Evangelisches Krankenhaus Hamm, Hamm, Germany; 27 Dept. Hematology and Oncology, St. Hedwig Krankenhaus Berlin, Berlin, Germany; 28 Dept. Hematology/Oncology, St. Antonius Krankenhaus Eschweiler, Eschweiler, Germany; 29 Dept. Medicine I, Klinikum Frankfurt/Oder, FrankfurtOder, Germany; 30 Dept. Internal Medicine II, Krankenhaus Martha-Maria, Halle, Germany; 31 Dept. Hematology, Oncology and Palliative Care, St. Marien-Krankenhaus Siegen, Siegen, Germany; 32 Dept. Hematology and Oncology, Katholisches Krankenhaus Hagen, Hagen, Germany; 33 Dept. Hematology and Oncology, Charité Campus Virchow, Berlin, Germany; 34 Institute of Biometry and Clinical Research, University Hospital Münster, Münster, Germany; 35 IIIrd Medical Dept, University Hospital of Mannheim, Mannheim, Germany; 36 IIIrd Medical Dept, University Hospital Großhadern, Munich, Germany; 37 Division of Hematology, Oncology and Tumour Immunology, Department of Medicine, Charité-Universitätsmedizin Berlin, Berlin, Germany; 38 Deutsche Gesellschaft für Hämatologie und Medizinische Onkologie, Berlin, Germany; 39 Dept. of Medicine A, University Hospital of Münster, Münster, Germany; 40 Dept. of Medicine V, University Hospital of Heidelberg, Heidelberg, Germany; 41 Department of Medicine, Hematology/Oncology, Goethe University, Frankfurt, Germany; 42 Mannheim, University of Heidelberg, Mannheim, Germany; 43 European LeukemiaNet, Weinheim, Germany; 44 Dept. of Medicine 3, Klinikum Leverkusen, Leverkusen, Germany

**Keywords:** Acute myeloid leukemia, Prognostic factors, Induction therapy, Complete remission, Consolidation therapy, Allogeneic stem cell transplantation

## Abstract

A randomized inter-group trial comparing more intensive treatment strategies to a common standard arm 3 + 7 (CSA) was conducted in patients with non-M3 AML. Untreated patients ≥ 60 years were allocated to the CSA (*n* = 132) or to the study group arms (*n* = 1154) of the AMLCG (TAD/HAM versus HAM/HAM ± G-CSF followed by TAD and maintenance) and the OSHO (intermediate-dose ara-C/mitoxantrone followed by ara-C/mitoxantrone). Median age of the 1147 eligible patients was 69 (range 60–87) years. CR/CRi status at 90 days was not significantly different between the CSA (54% (95%CI: 45–64)) and the study group arms (53% (95%CI: 47–60) and 59% (95%CI: 58–63)). The five-year event-free survival (EFS) probability (primary endpoint) was 6.2% (95%CI: 2.7–14.0) in the CSA, 7.6% (95%CI: 4.5–12.8) in study group A and 11.1% (95%CI: 9.0–13.7) in B. The 5-year OS was 17.2% (95%CI: 11.0–26.9), 17.0% (95%CI: 2.0–23.9), and 19.5% (95%CI: 16.7–22.8) in CSA, study group A and B, respectively. Neither study group differed significantly from the CSA regarding EFS, OS, or relapse-free survival. In multivariate analyses, allocation to the treatment strategy was not significantly associated with the time-to-event endpoints. The evaluation of more intensive treatment strategies did not show clinically relevant outcome differences when compared to CSA.

**Supplementary Information:**

The online version contains supplementary material available at 10.1007/s00277-023-05087-8.

## Introduction

Almost 140,000 cases of acute myeloid leukemia (AML) and 100,000 deaths are reported worldwide per year with steady increasing incidence largely due to population growth and aging [1]. While progress has been made particularly in younger patients through intensive chemotherapy (IC) and stem cell transplantation (HSCT), the majority of AML patients (> 60 years of age) have historically been considered to be ineligible for intensive therapies because of comorbidities, more aggressive leukemia biology, and reduced tolerance to intensive therapy. On the other hand, IC in newly diagnosed elderly AML patients, with or without poor performance status, does improve survival when compared to best supportive care [2, 3]. A randomized study of IC vs. best supportive care combined with mild cytoreductive therapy confirmed better survival by IC with comparable hospitalization frequency [4]. Despite these findings, the majority of older patients with AML are not offered IC and those receiving it had a 5-year survival rate of only 8% [5, 6]. More recently, treatment rates have increased from 35 to 50% following improvements in supportive therapy [7, 8]. Elderly patients are now treated similarly to younger patients with the aim of inducing complete remission (CR) and maintaining long-term remission using consolidation and/or HSCT. Although inferior to results in younger patients, CR rates have improved up to 66.7% [9].

Recent discoveries in biology have enriched treatment options for AML. Modifying epigenetics with hypomethlating agents (HMA) induces CRs with lower toxicity than IC in some pretreated patients and patients with comorbidities [10]. In addition to epigenetics, disturbance in the regulation of apoptosis involving, e.g., bcl-2 has been identified as common mechanism in AML. The concept of blocking bcl-2 has been tested successfully in refractory disease as monotherapy and in combination with epigenetic therapy in newly diagnosed patients with AML [11]. These treatments lead to CR rates similar to those of IC with a high proportion of molecular remissions and low therapy-related mortality [12, 13].

Inhibition of driver mutations or their products in sub-groups of newly diagnosed patients with AML has increased in combination with chemotherapy overall survival [14]. Other targeted therapies such as IDH inhibitors have shown promising results as mono- or combination therapies in phase I and II studies [15–18].

Many of these new treatment approaches are now being tested in combination with IC as first line therapy, which remains the backbone of therapy even in fit elderly patients. The situation is further complicated by selection bias for eligibility to IC due to increased disease risk and comorbidities. Furthermore, because of disease heterogeneity, determining outcome of low, intermediate, and poor risk disease may be of crucial importance for choosing the best treatment intensity and strategy.

The ideal IC aims to balance between efficacy and therapy-induced morbidity and mortality without selection bias and still needs to be defined. For this reason, we considered the well-established standard 3 + 7 protocol as baseline and compared the outcome to those of patients treated with more intensive treatment regimens of two AML German study groups [12, 19, 20]. A randomization ratio of 9:1 was chosen to allow study group specific questions to be answered.

## Patients and methods

### Patients

Patients ≥ 60 years of age with non-promyelocytic AML were centrally randomized up-front in a 9:1 assignment to study specific arms of the German AML cooperative Group (AMLCG) or the East German Study Group Hematology and Oncology (OSHO) compared to a CSA (suppl. Figure S1). The AMLCG study arm randomized TAD (ara-C 100 mg/m^2^/d continuous infusion (CI) d1-2 followed by 30-min IV infusion BID d 3–8, daunorubicin 60 mg/m^2^/d IV d 3–5 and 6-thioguanine 100 mg/m^2^/d p.o. BID d 3–9) followed by HAM (ara-C 1 g/m^2^/d IV BID d 1–3 and mitoxantrone 10 mg/m^2^/d IV d 3–5) versus two courses of HAM ± G-CSF, with the second induction course only applied in case of blast persistence. One course of TAD was given as consolidation followed by maintenance chemotherapy over three years [21]. The OSHO AML04 study included ara-C 1 g/m^2^/d BID IV d 1 + 3 + 5 + 7 and mitoxantrone 10 mg/m^2^/d IV d 1 – 3 for one or two induction courses and ara-C 500 mg/m^2^ BID 1 h IV d 1 + 3 + 5 in combination with mitoxantrone 10 mg/m^2^/d IV d 1 + 2 as consolidation twice. Pegfilgrastim 6 mg s.c. was given on day 10 of induction and on day 8 of consolidation. Allogeneic related or unrelated HSCT following non-myeloablative conditioning was considered after CR. The CSA consisted of one or two induction cycles of ara-C 100 mg/m^2^/d CI d 1–7 and daunorubicin 60 mg/m^2^/d IV d 3, 4, 5 (3 + 7 regimen) followed by two courses of ara-C 1 g/m^2^/d BID IV d 1 + 3 + 5 as consolidation [20]. Detailed information on therapies of the study groups and CSA are given in suppl. Figure 1. Cytogenetic and molecular risk was determined as previously described [22].

Inclusion criteria contained all consecutive AML (de novo, secondary, and therapy related, except APL) diagnosed in the study period. Exclusion criteria included inability of the patient to understand the study and give informed consent, non AML-related renal insufficiency, liver insufficiency, cardiac insufficiency NYHA III + IV, concurrent acute myocardial infarction, and uncontrolled infection such as pneumonia with hypoxia or septic shock.

The study was approved by the Institutional Review Board (IRB) of the University of Leipzig, registered at clinicaltrials.gov (NCT01497002 and NCT00266136) and the approval notified to IRBs of the participating centers. Patients had given written informed consent prior to study enrollment and randomization.

### Definitions and statistical considerations

The primary endpoint of the study was event-free survival (EFS events: no CR/no CR with incomplete hematological recovery (CRi) 90 days after start of therapy, relapse, or death). Secondary endpoints were CR/CRi rate, overall survival (OS, event: death), and relapse free-survival (RFS events: relapse or death). Apart from CR/CRi, patient status 90 days after start of therapy comprised persistent leukemia (≥ 5% blasts after induction therapy), early death (up to 1 week after the end of the first course of induction), death in hypoplasia (death > 1 week after end of first induction treatment in hypoplasia and < 5% blasts), or death from indeterminate cause in case of unknown presence of AML. Apart from the primary endpoint analysis, all other analyses including non-relapse-mortality (NRM, event: death in first CR/CRi) and relapse incidence (RI, event: relapse) were explorative and without adjustment for multiple testing.

CR, CRi, and relapse were defined as published previously [22]. EFS and OS were measured from start of therapy until an event was observed. RFS, NRM, and RI were defined as time from CR/CRi to observation of their corresponding events. For patients without an event, all survival endpoints were censored at the date of last follow-up.

The aim of the study was to compare the common standard arm with each study group on its own. Different group-specific arms within a study group were not considered. This was left to the study group internal analysis. Instead, the results of the common standard arm were compared with the results of the general treatment concept of each study group. Thus, no formal test of interaction was performed. Differences in baseline characteristics between the standard arm and the study group arms were investigated by Fisher’s exact test, the Wilcoxon-Mann–Whitney U test, or the Cochran-Armitage trend test [23] as appropriate. Unadjusted probabilities of OS, EFS, and RFS were calculated by the Kaplan–Meier method. To adjust for variations in baseline characteristics with prognostic influence, differences between the survival probabilities of the standard treatment arm and any of the studies’ own groups were judged in a multiple Cox regression model [24] by the Wald test with all influential co-variables included. In addition, direct adjusted survival curves based on the Cox regression model with all influential co-variables stratified for the studies were estimated [25]. Regarding the achievement of CR/CRi after induction therapy, adjustment for prognostic variables was performed through multiple logistic regression [26]. NRM and RI were calculated via cumulative incidence in a competing risk setting, the competing risk being relapse before death for NRM and death in first CR/CRi for RI. For NRM and RI, differences between treatment strategies were assessed utilizing the Fine and Gray model with all significant prognostic co-variables included [27].

With respect to the primary endpoint, the null hypotheses were that there would be no difference in the EFS probabilities when the intergroup arm was compared to study group A or to study group B. For each of the two tests, the overall significance level of 0.05 was allowed since data of study group A and study group B came from two independent studies and were not used within the same test. All *p* values are two-sided. Regarding the primary end point comparisons of each study with the according results of the standard treatment, the group sequential design of O’Brien-Fleming [28] with three interim analyses was applied, allowing *α* = 0.04291 for the final analyses. For final decisions on significance with regard to the comparisons between the standard arm and each study’s treatment strategy, *p* values of the adjusted multiple regression analyses for OS, EFS, RFS, CR/CRi, NRM, and RI were preferred over those of the unadjusted analyses (log-rank test for OS, EFS, and RFS; Fisher’s exact test for CR/CRi, Gray test for NRM and RI). Use of adjusted analyses was not pre-specified in the protocol for both the primary and secondary outcome measures, but deemed preferable due to differences in prognostic factors. However, use of unadjusted analyses did not lead to changes in significance. All analyses were performed with the SAS software version 9.4 (SAS Institute, Cary, NC), all graphical outputs were created using R version 4.1.0 (R Core Team 2013).

## Results

**Fig. 1:**
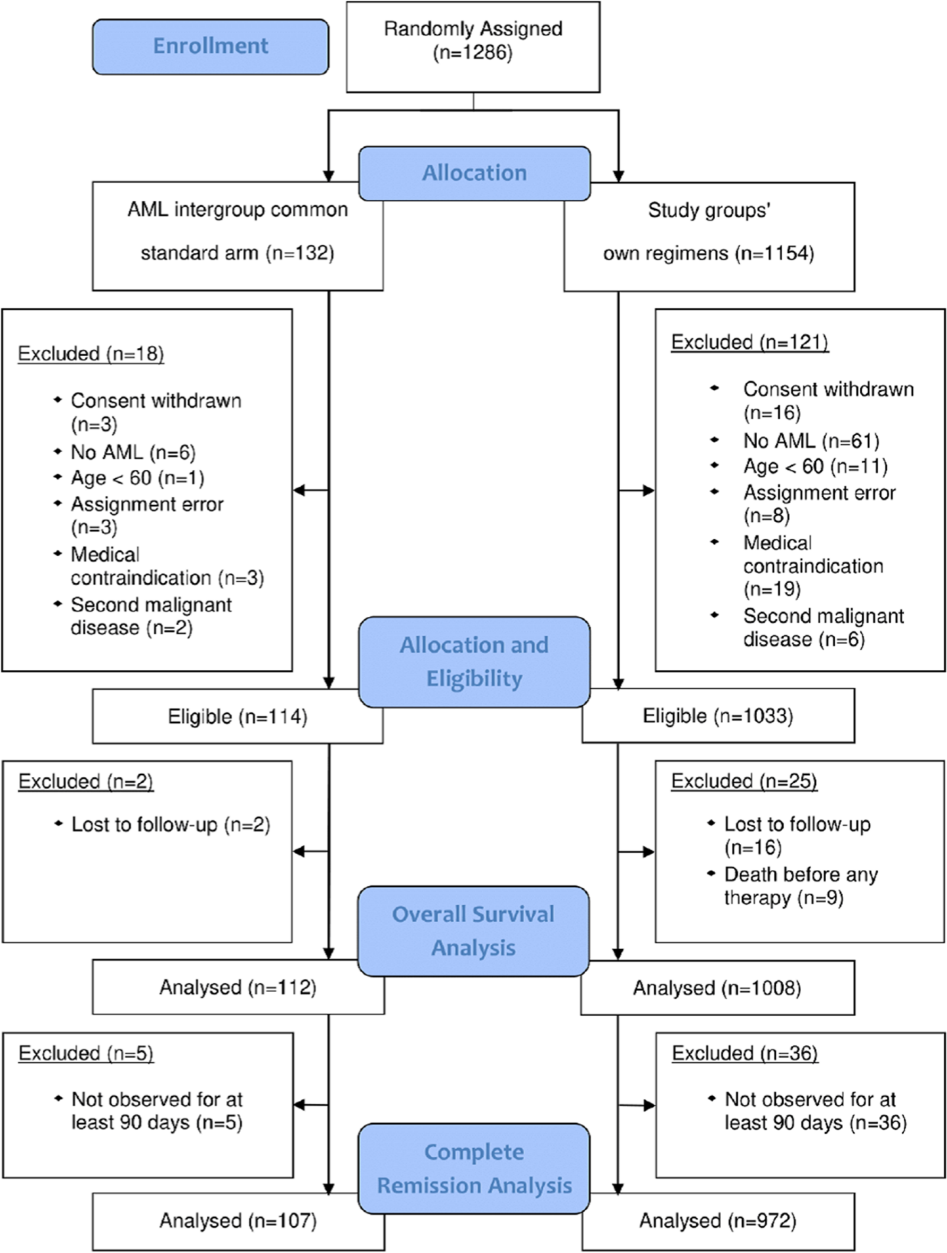
Consort flow diagram. Allocation of AML patients to the arms, eligibility, CR and overall survival analyses

Between April 1, 2005, and May 26, 2015, 1286 patients were randomly assigned to the CSA (*n* = 132) or to the study groups arms (*n* = 1154; Fig. 1). After excluding 139 patients (10.8%) due to in- and exclusion criteria violation, 1147 patients were eligible for analysis (114 of them (9.9%) assigned to the CSA). A total of 1120 patients had follow-up for OS and 1079 patients were available for CR analysis (Fig. 1). Baseline characteristics of all eligible patients showed median ages of 68 (range 60–82) years for the CSA, 70 (60–85) years for study group A, and 69 (60–87) years for the study group B (Table 1). The CSA had a significantly different molecular marker distribution compared with study A (*p* = 0.04), but not with study B. No significantly different distributions were found with respect to the proportions of patients with secondary AML, cytogenetic risk groups, white blood cell counts, and LDH.

**Table 1:** Patient characteristics according to the allocation to common standard arm (CSA), study group A and B

	Common standard arm		Study group A		Study group B		*p* value	
	*n*	%	*n*	%	*n*	%	A vs. CSA	B vs. CSA
Eligible patients	114		223		810			
Age; median (range) years	68 (60–82)		70 (60–85)		69 (60–87)		0.07	0.74
Female (*n*)	47	41	102	46	370	46	0.48	0.42
Secondary AML (*n*)	41	36	67	30	343	43	0.32	0.22
WBC count (*n*)	113		199		796		0.88	0.91
Median (range) 10^9^/L	6.8 (0.6–349)		6.6 (0.4–300)		6.9 (0.2–450)			
Lactate dehydrogenase (*n*)	108		191		758		0.67	0.40
Median (range) U/L	365 (106–7260)		301 (96–8713)		370 (51–7002)			
Cytogenetic group (*n*)	99	97	202	91	658	81	0.44	0.86
Favorable^*^	10	10	17	8	90	14		
Intermediate^**^	68	69	135	67	397	60		
Adverse	21	21	50	25	171	26		
Normal cytogenetics	29	29	72	36	242	30	0.21	0.38
Molecular marker	29	25	72	32	242	30	**0.04**	0.09
NPM1 wild type and no FLT3-ITD	9	31	42	58	124	51		
NPM1 wild type and FLT3-ITD	5	17	4	6	22	9		
NPM1 mut and no FLT3-ITD	9	31	13	18	69	29		
NPM1 mut and FLT3-ITD	6	21	13	18	27	11		

*CSA*, common standard arm; *A*, study group A; *B*, study Group B. ^*^Includes favorable cytogenetics and normal karyotype with nucleophosmin gene (NPM1) mutation and no fms-related tyrosine kinase 3 gene internal tandem duplications (FLT3-ITD). Apart from that, cytogenetic classification of all three group was in accordance with Döhner et al. [13]. ^**^Includes intermediate cytogenetics and normal karyotype with wild-type NPM1 mutation or with FLT3-ITD (intermediate 1 and 2)^13^. Values in bold indicate statistically significance

### Outcome

After 90 days of therapy, 54.0% (95% CI: 45–64) of the patients in the CSA had achieved CR or CRi, which barely differed from the results of the study groups’ own regimens (study group A 53% (95% CI: 47–60) and study group B 59% (95% CI: 56–63); Table 2). Adjusting the comparisons CSA vs. group A and CSA vs. group B by including the significant prognostic variables cytogenetic/molecular risk group, type of disease at diagnosis, WBC, and age in a common logistic regression model, no significant differences between the CR/CRi rates were identified. Overall death rate at 90 days was not significantly different between the CSA (24%) and each of the study groups independently (27% study group A and 19% study group B, Table 2). Persistent leukemia at day 90 was noted in 16% of the standard arm as compared to 12% and 17% in the two study group arms, respectively.

**Table 2:** Clinical course of patients after treatment in the common standard arm (CSA), arm A and B

	Common standard arm		Study group A		Study group B	
	No	% (95% CI)	No	% (95% CI)	No	% (95% CI)
No. of evaluable patients	107		199		773	
CR/CRi	62	54 (45–64)	119	53 (47–60)	479	59 (56–63)
Hypoplasia without persistent AML	7	7 (3–13)	23	12 (8–17)	92	12 (10–14)
Persisting disease at 90 days	17	16 (10–24)	23	12 (8–17)	129	17 (14–19)
Relapse at 90 days	2	2 (1–7)	3	2 (1–4)	19	2 (1–4)
Death without AML within 90 days	3	3 (1–8)	5	3 (1–6)	18	2 (1–4)
Death with AML within 90 days	10	9 (5–17)	9	5 (2–8)	43	6 (4–7)
Death from indeterminate cause within 90 days	13	12 (7–20)	40	20 (15–26)	85	11 (9–13)
Death within 90 days	26	24 (16–32)	54	27 (21–33)	146	19 (16–22)
EFS %(CI %) at 5 years		6.2 (2.7–14.0)		7.6 (4.5–12.8)		11.1 (9.0–13.7)
OS %(CI %) at 5 years		17.2 (11.0–26.9)		17.0 (12.0–23.9)		19.5 (16.7–22.8)
RFS %(CI %) at 5 years		13.8 (7.3–25.9)		14.6 (9.2–23.1)		20.6 (17.1–24.8)
RI %(CI %) at 5 years		74.9 (61.9–84.1)		65.0 (55.3–73.1)		61.0 (56.4–65.3)
NRM %(CI %) at 5 years		11.3 (5.2–20.0)		20.4 (13.7–28.0)		18.4 (15.0–22.0)

*CSA*, common standard arm; *CR/CRi*, complete or incomplete remission; *EFS*, event free survival; *OS*, overall survival; *RFS*, relapse free survival; *RI*, relapse incidence; *NRM*, non-relapse mortality

**Fig. 2:**
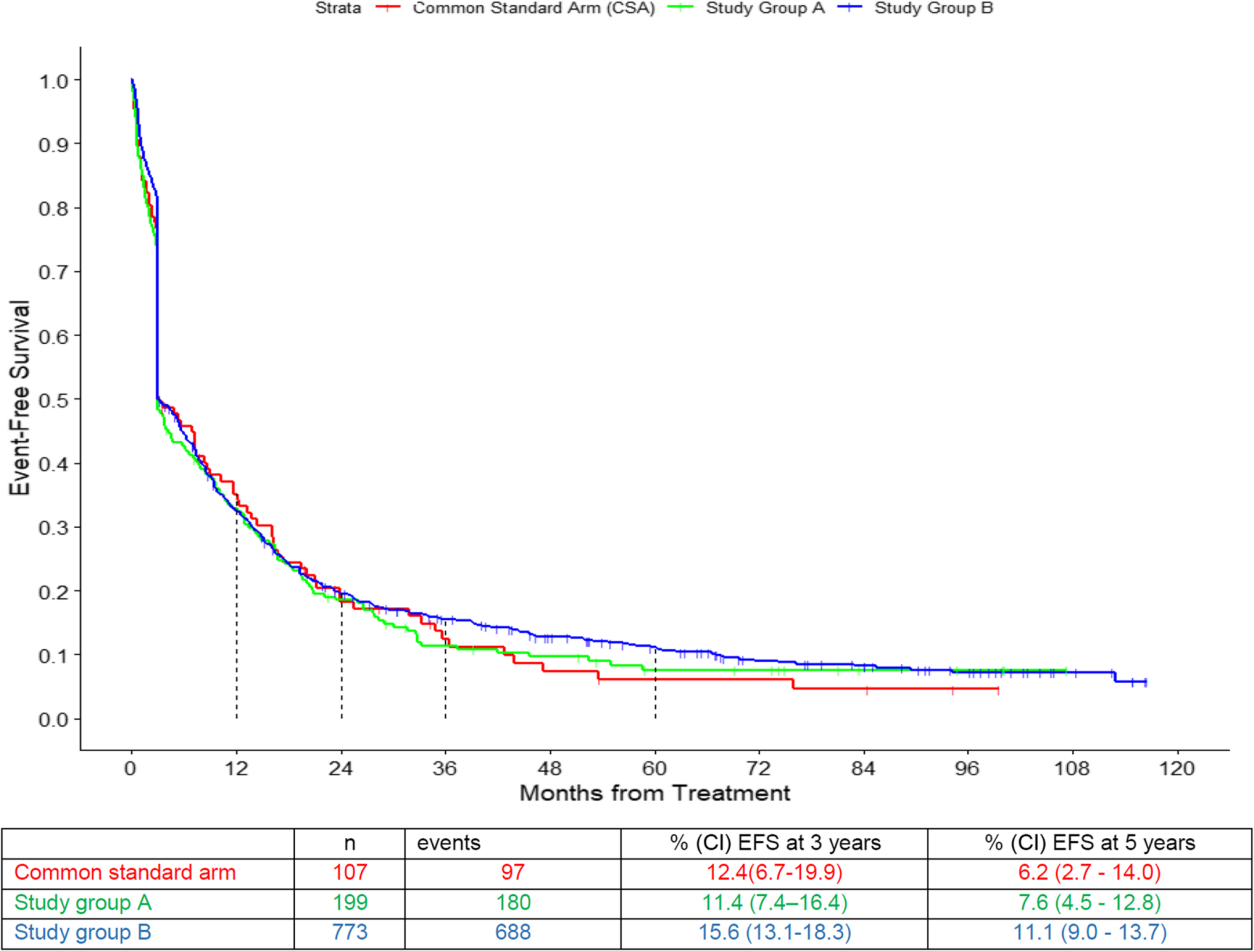
Event-free survival (EFS) of the three arms: common standard arm (CSA), study group A, and study group B

The probabilities for EFS between the CSA and the two study group regimens (primary endpoint) did not differ significantly (Table 2, Fig. 2). Five-year EFS was 6.2% (95% CI: 2.7 – 14.0) in the CSA, 7.6% (95% CI: 4.5 – 12.8) in study A, and 11.1% (95% CI: 9.0 – 13.7) in study B. In the multivariate analysis age, type of disease, cytogenetic group, and WBC count at diagnosis were independent prognostic factors, but treatment group was not (Table 3).

**Table 3:** Multi-variable Cox-PH regression to identify variables with influence on EFS, OS, and RFS

	EFS					OS					RFS				
Variable	*n*	Events	Estimated coeff. ± SD	Estimated HR 95% CI	*p* value	*n*	Events	Estimated coeff. ± SD	Estimated HR 95% CI	*p* value	*n*	Events	Estimated coeff. ± SD	Estimated HR 95% CI	*p* value
Treatment group															
CSA	92	82	Baseline			97	72	Baseline			61	48	Baseline		
Study group A	167	152	0.04 ± 0.14	1.04 (0.80–1.37)	0.75	179	135	0.04 ± 0.15	1.04 (0.78–1.39)	0.78	109	87	-0.01 ± 0.18	0.98 (0.69–1.40)	0.93
Study group B	635	596	-0.11 ± 0.12	0.90 (0.71–1.13)	0.37	647	514	-0.06 ± 0.13	0.94 (0.73–1.20)	0.61	428	334	-0.19 ± 0.15	0.83 (0.61–1.13)	0.23
Significant variables in the final model															
Age, years/10	894	803	0.29 ± 0.07	1.34 (1.16–1.53)	< **0.0001**	923	721	0.45 ± 0.07	1.57 (1.36–1.82)	**< 0.0001**	598	469	0.36 ± 0.10	1.43 (1.19–1.73)	**0.0001**
Type of disease															
De novo AML	544	475	Baseline			558	417	Baseline			386	294	Baseline		
Secondary AML	350	328	0.31 ± 0.07	1.37 (1.19–1.58)	**< 0.0001**	365	304	0.27 ± 0.08	1.30 (1.12–1.52)	**0.0006**	212	175	0.12 ± 0.09	1.13 (0.94–1.37)	0.21
Cytogenetic risk group					**< 0.0001**					**< 0.0001**					**< 0.0001**
Favorable	111	85	Baseline			113	73	Baseline			90	59	Baseline		
Intermediate	561	505	0.57 ± 0.12	1.77 (1.40–2.23)		583	446	0.48 ± 0.13	1.62 (1.26–2.08)		372	289	0.44 ± 0.14	1.55 (1.17–2.06)	
Adverse	222	213	0.91 ± 0.13	2.48 (1.91–3.22)		227	202	0.94 ± 0.14	2.55 (1.93–3.36)		136	121	0.94 ± 0.17	2.57 (1.86–3.56)	
Log ((WBC in 10^9^/L)/10)	894	803	0.09 ± 0.02	1.10 (1.05–1.15)	** < 0.0001**	923	721	0.09 ± 0.02	1.09 (1.04–1.15)	**0.0002**	598	469	0.04 ± 0.03	1.04 (0.98–1.11)	0.17

*CSA*, common standard arm; *Coeff.*, coefficient; *SD*, standard deviation; *HR*, hazard ratio; *EFS*, event free survival; *OS*, overall survival; *RFS*, relapse free survival. For age and WBC, the same variable transformations as identified in Büchner et al. [29] were chosen. Values in bold indicate statistically significance

**Fig. 3:**
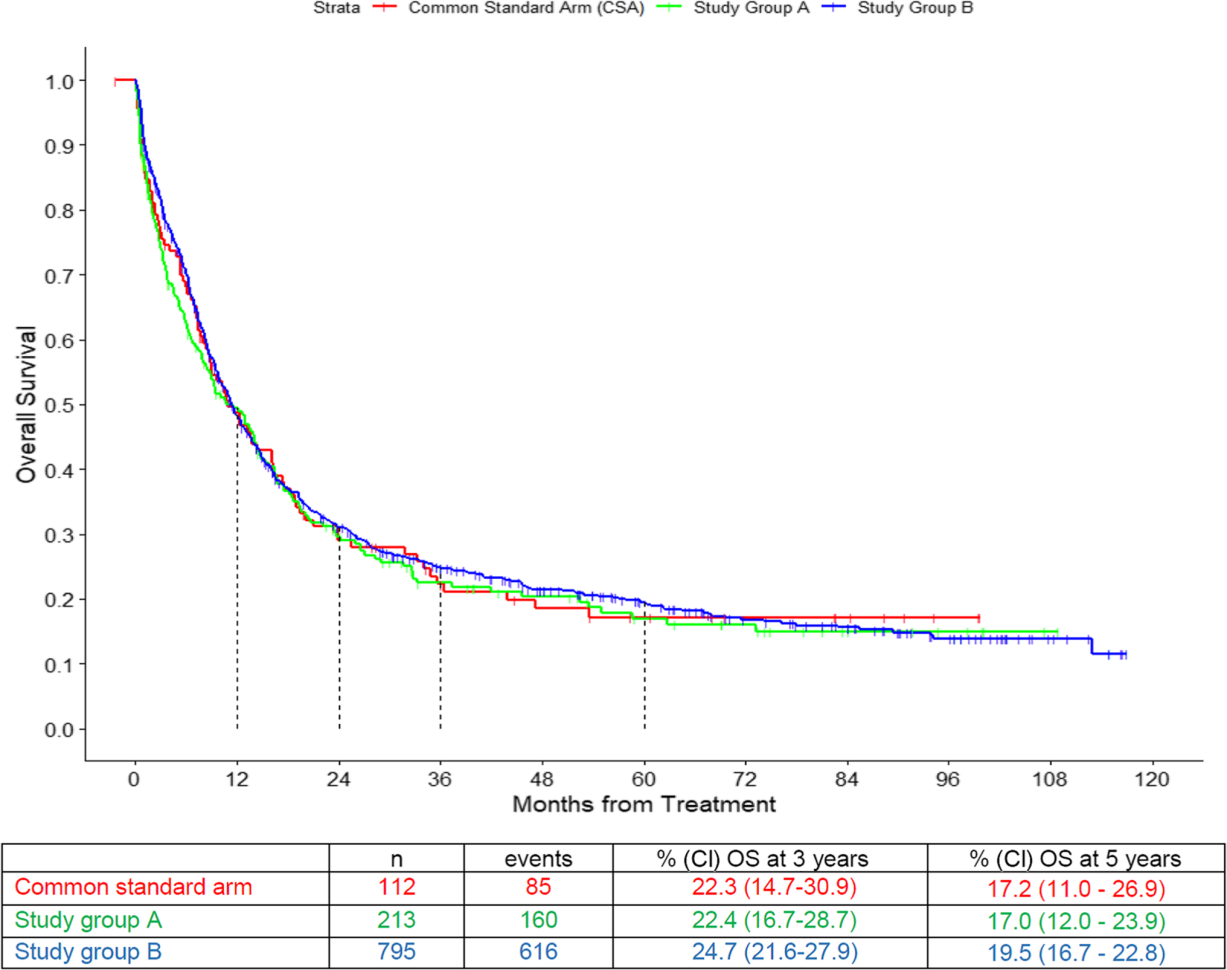
Overall survival (OS) of the three arms: common standard arm (CSA), study group A, and study group B

Median observation time was 67 months. OS did not differ significantly between the CSA and the study groups’ own regimens (Fig. 3). The 5-year survival probability was 17.2% (95% CI: 11.0–26.9) in the CSA, 17.0% (95% CI: 12.0–23.9) in the study group A, and 19.5% (95% CI: 16.7–22.8) in the study group B. Study group affiliation was not significant for OS, in contrast to age, type of disease, cytogenetic risk group, and WBC at diagnosis (all *p* < 0.0001; Table 3).

**Fig. 4:**
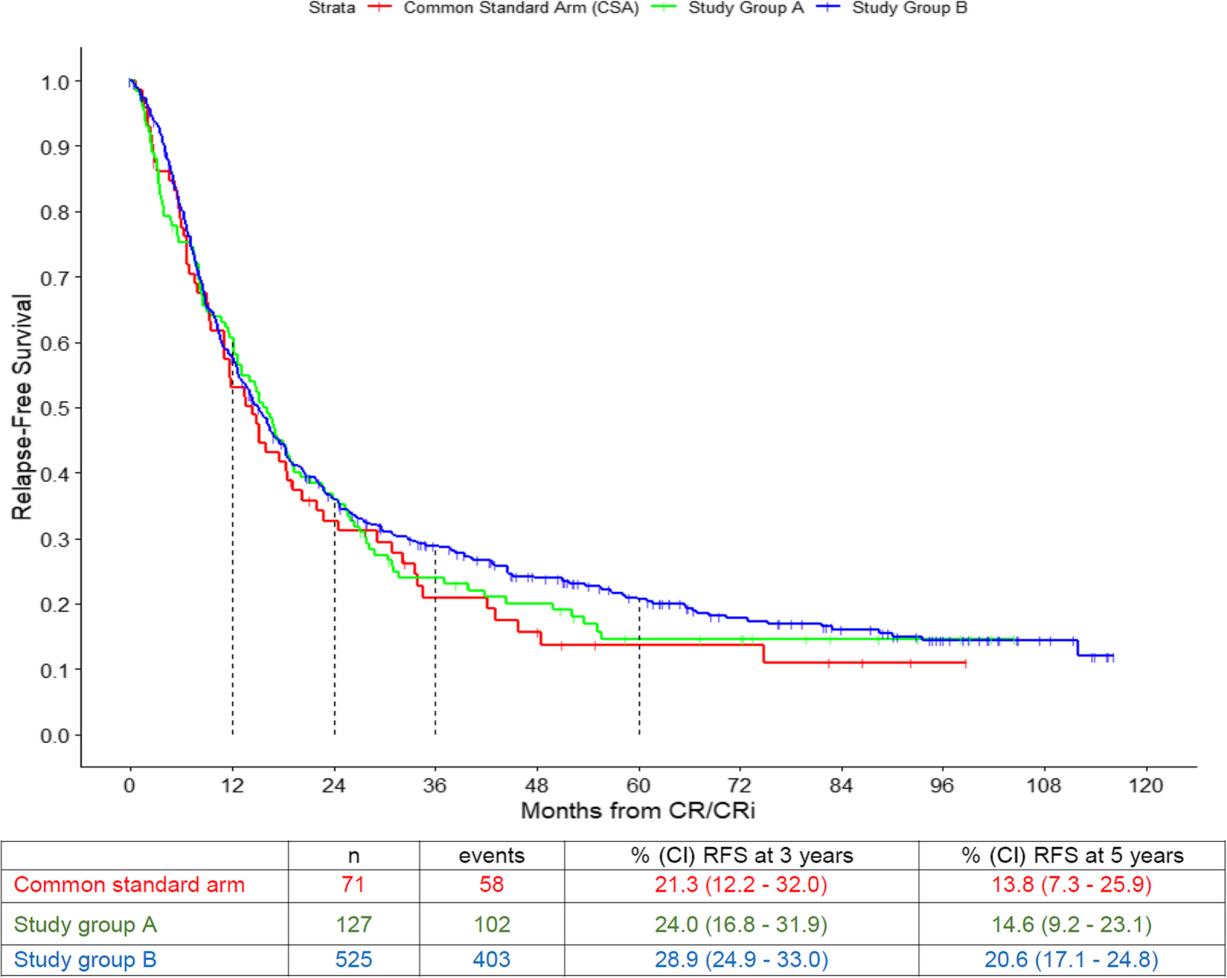
Relapse-free survival (RFS) of the three arms: common standard arm (CSA), study group A, and study group B

The 5-year RFS probability was 13.8% (95% CI: 7.3 – 25.9) in the CSA, 14.6% (95% CI: 9.2 – 23.1) in arm A, and 20.6% (95% CI: 17.1 – 24.8) in arm B without significant differences (Fig. 4). In the Cox model only age and cytogenetic risk were statistically significant for RFS, treatment group was not (Table 3).

**Fig. 5:**
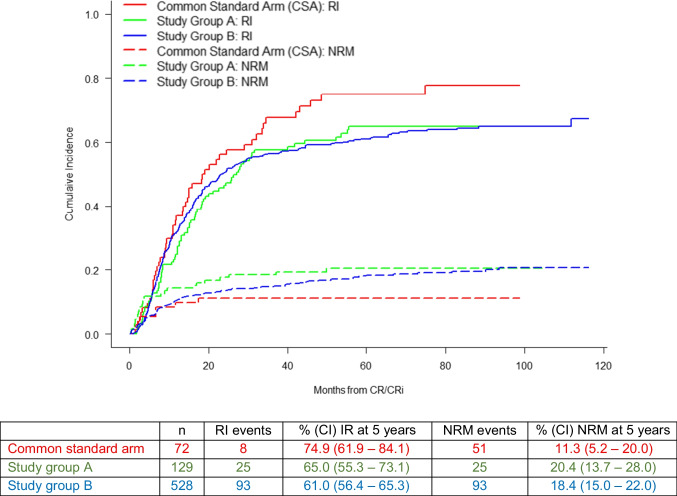
Non-relapse mortality (NRM) and relapse incidence (RI) of the three arms: common standard arm (CSA), study group A, and study group B

To adjust survival probabilities of the treatment groups by the significant covariates identified in the respective Cox model, adjusted EFS, OS, and RFS probabilities were computed (suppl. Figure S2, S3 and S4). NRM and RI were estimated, but no statistically significant differences between the treatment groups were observed (Fig. 5). At 5 years, RI amounted to 74.9% (95% CI: 61.9 – 84.1) in the CSA, 65% (95% CI: 55.3 – 73.1) in study A, and 61.0% (95% CI: 56.4 – 65.3) in study B. NRM was calculated for the same patient collective, revealing 5-year NRM rates of 11.3 (95% CI: 5.2 – 20.0) in the CSA, 20.4 (95% CI: 13.7 – 28.0) in arm A, and 18.4 (95% CI: 15.0 – 22.0) in arm B.

## Discussion

The most widely utilized intensive induction chemotherapy for AML was first published in 1973 [30] and, after further refinements in the 80 s, has been used in the current form ever since [19, 31]. Over this period, a number of clinical trials have investigated induction intensity following dose dependent efficacy concepts, new drug combinations, and sequential therapies. Somewhat surprisingly, a prospective intergroup analysis in younger (< 60 years) patients with AML compared protocols of differing intensities from five German study groups against a CSA and did not find any statistical significant difference in outcomes [29].

Until two decades ago, it was generally accepted that elderly patients with AML should not be treated intensively because of adverse biology, comorbidities, and dismal survival. This attitude changed after long-term survival was observed in a small proportion of elderly patients after IC and results improved to 19.5% OS at 5 years. In the current randomized inter-group trial, we aimed to focus on induction intensity in patients ≥ 60 years of age by analyzing treatment concepts of different intensities within two German study groups compared to the standard 3 + 7 protocol (CSA) [16]. Comparison of the treatment strategies did not show clinically relevant outcome differences when compared to the CSA in CR rate, EFS, OS, and RFS. The study groups had lower RI, but these differences were not statistically significant and counteracted by a numerical higher NRM, again with no significant difference. Risk factors for EFS and OS identified in the patients included age, type of disease, cytogenetic risk group, and WBC counts at diagnosis, but not treatment strategy.

The results described in this study are of importance for several reasons. First, efficacy results showed no significant difference between either intensified induction and the established 3 + 7 protocol. This protocol continues to be the reference for further studies exploring combinations with targeted therapies. Second, the results of this multi-center intergroup study suggest improved EFS (6.2%, 7.6%, and 11.1%) and OS probabilities (17.0%, 17.2%, and 19.5%) at 5-years in patients with AML ≥ 60 years as compared with historical controls (OS 8% at 5 years) [6]. This may result from better supportive therapy and standardized clinical management. Third, this trial confirms the feasibility of IC in elderly patients up to 87 years. Age itself influenced EFS and OS, as did cytogenetic risk group and WBC at diagnosis. Finally, AML persistence rates after ≤ 2 induction cycles were higher in this population (14.7%) than in younger patients (7.6%) [9] and the death rate in the first 90 days with and without leukemia was 20.9%. In addition, NRM and RI were not statistically significant different between the treatment groups.

The randomized design with broad inclusion criteria and a large number of patients is a particular strength of this multi-center study and provides real world information. Our estimation of patients not included in this study is in the range of 20%, which is a clear improvement on previous figures of 35–50% [7, 8]. Potential weaknesses include the small sub-groups of very high risk AML patients (e.g., TP53 mutated), which may show differential response to different treatment strategies. A further limitation of this analysis is the restriction to prognosis based on cytogenetic risk and to FLT3-ITD and NPM1 mutations only, since additional molecular features at diagnosis are unavailable. Furthermore, it is not possible to evaluate the effect of HSCT due to the fact that only a small proportion received this treatment. Randomized studies of the role of HSCT are currently under evaluation [32].

New concepts are needed to further improve the results for elderly patients with AML. Obtaining higher CR rates and increasing the depth of CR might be one way to reach this goal. The use of new delivery formulations such as the liposomal formulation (e.g., CPX-351) may be one way of increasing the efficacy of induction chemotherapy [33, 34].

The importance of epigenetic changes in the initiation of AML has been discovered during the last decade and hypomethylating agents (HMA) are increasingly used in patients not eligible for IC and in elderly patients [10, 35]. Although not curative, HMA are able to induce CRs even in pretreated patients and patients with comorbidities and display lower toxicity than IC [10]. This has prompted the practice of response adapted sequential therapy in elderly patients with AML using HMA initially and then IC in non-responding patients [36]. The results of this study are currently awaited. Further discoveries in the biology of AML are opening new frontiers. In addition to the role played by epigenetic changes, disturbance in the regulation of apoptosis involving bcl-2 have been identified as important common mechanism in AML. The concept of blocking bcl-2 has been tested successfully in refractory disease as monotherapy and in combination with epigenetic therapy in newly diagnosed patients with AML [11]. These treatments lead to CR rates similar to those of IC with a high proportion of measurable residual disease negative patients and low therapy-related mortality [12, 13]. Randomized studies will show if IC can be replaced either by this combination or even by triple induction therapies to induce CR in patients with AML. Results to date suggest that some responses may be short lived and that development of resistance is the limiting factor for long-term remission. Improvements in consolidation therapy and/or maintenance therapy may be one solution for avoiding relapse caused by resistance to these drugs or drug combinations.

Inhibition of activating driver mutations increases the treatment options in AML. Inhibitors of FLT3 mutations, which are now available with various specificity and potency characteristics, have been studied in the context of both mono- and in combination therapy. The addition of TKI to chemotherapy has been shown to increase overall survival and has been approved for newly diagnosed patients [14]. The potential of second generation TKI to induce CR as a low toxicity monotherapy has been tested in relapsed and refractory patients [37, 38]. Second generation FLT3 inhibitors are currently tested in combination to IC and are expected to further improve results in newly diagnosed patients. While clearly being of high interest, this approach is restricted to the 1/3 of all AML patients who have FLT3 mutated disease and often results in resistance or relapse that limit long term remissions. Other targeted therapies such as IDH inhibitors have shown promising results as mono- or combination therapies in phase I and II studies [15–18].

Meanwhile, the determination of measurable residual disease is enabling quantification and monitoring of the depth of response either by molecular or flow cytometry determination methods. This will allow better evaluation of CR and management of personalized therapy. Reducing treatment related mortality may be another approach to improve outcome of elderly patients with AML.

Finally, the use of HSCT following reduced or non-myeloablative conditioning to decrease the relapse risk is another promising approach. Such protocols have been successfully established in patients up to 75 years and older [39–41]. Other consolidation or maintenance therapies including immunological concepts to eradicate the malignant stem cell or clones are being investigated.

In conclusion, more intensive treatment strategies did not show clinically relevant outcome differences when compared to CSA, but an overall long-term improvement compared to previous publications in patients ≥ 60 years with newly diagnosed AML. Intensive chemotherapy remains the backbone for long term survival. The outcome of this clinical trial provides an important contribution for the selection of IC to be used in combination with targeted or new treatment modalities in future studies involving treatment naive AML patients. In addition, it proves that an innovative trial design, like in our study, may help answering important clinical questions without hampering study group specific questions.

## Supplementary Information

Below is the link to the electronic supplementary material.

Supplementary file1 (DOCX 112 KB)

## Data Availability

Dataset can be accessed in anonymized form at the Institut für Medizinische Informationsverarbeitung, Biometrie und Epidemiologie (IBE), Ludwig Maximilian Universität München, Germany.
