# Supplementary material for: Different treatment strategies versus a common standard arm (CSA) in patients with newly diagnosed AML over the age of 60 years: a randomized German inter-group study
Source: Ann Hematol. 2023 Jan 25;102(3):547–61. doi: 10.1007/s00277-023-05087-8 (PMC9977880; doi:10.1007/s00277-023-05087-8)
Supplement: Supplementary file 1 — Supplementary file1 (DOCX 112 KB) [file 277_2023_5087_MOESM1_ESM.docx]

**Supplements**

**Legend to the suppl. Table**

**Suppl. Table S1: Multivariate logistic regression for CR/CRi**

**Legend to the suppl. Figures**

**Suppl. Figure S1: Design of the studies and treatment characteristics**

**Suppl. Figure S2 : Standardized event-free survival (EFS) of the three arms: common standard arm (CSA), study group A and study group B**

**Suppl. Figure S3 : Standardized Overall survival (OS) of the three arms: common standard arm (CSA), study group A and study group B**

**Suppl. Figure S4 : Standardized relapsed-free survival (RFS) of the three arms: common standard arm (CSA), study group A and study group B**

| **suppl. Table 1: Multivariate logistic regression for CR/CRi** | | | | | |
| --- | --- | --- | --- | --- | --- |
|  | **CR/CRi** | | | | |
| **Variable** | **n** | **events** | **Estimated Coeff. ± SD** | **Estimated**  **OR 95% CI** | **p-value** |
| **Treatment group** | | | | | |
| CSA | 98 | 54 | Baseline |  |  |
| Study group A | 183 | 101 | 0.002 ± 0.26 | 1.00 (0.60-1.67) | .99 |
| Study group B | 650 | 393 | 0.24 ± 0.23 | 1.27 (0.81-1.97) | .29 |
| **Significant variables in the final model** | | | | | |
| **Age, years / 10** | 931 | 548 | -0.48 ± 0.14 | 0.62 (0.48-0.81) | **.0004** |
| **Type of disease** | | | | | |
| De novo AML | 564 | 360 | Baseline |  |  |
| Secondary AML | 367 | 188 | -0.59 ± 0.14 | 0.55 (0.42-0.73) | **<.0001** |
| **Cytogenetic risk group** | | | | |  |
| Favorable | 115 | 87 | Baseline |  |  |
| Intermediate | 587 | 341 | -0.91 ± 0.24 | 0.40 (0.25-0.65) | **.0002** |
| Adverse | 229 | 120 | -1.21 ± 0.27 | 0.29 (0.17-0.50) | **<.0001** |
|  | | | | | |
| **Log [(WBC in 10^9^/L) / 10]** | 931 | 548 | -0.19 ± 0.04 | 0.82 (0.75-0.89) | **<.0001** |
| CSA, common standard arm; CR/CRi, Complete or incomplete remission; Coeff., coefficient; SD, standard deviation; OR, odds ratio; EFS, event free survival; OS, overall survival; RFS, relapse free survival. For age and WBC, the same variable transformations as identified in Büchner et al. (2012) were chosen. | | | | | |


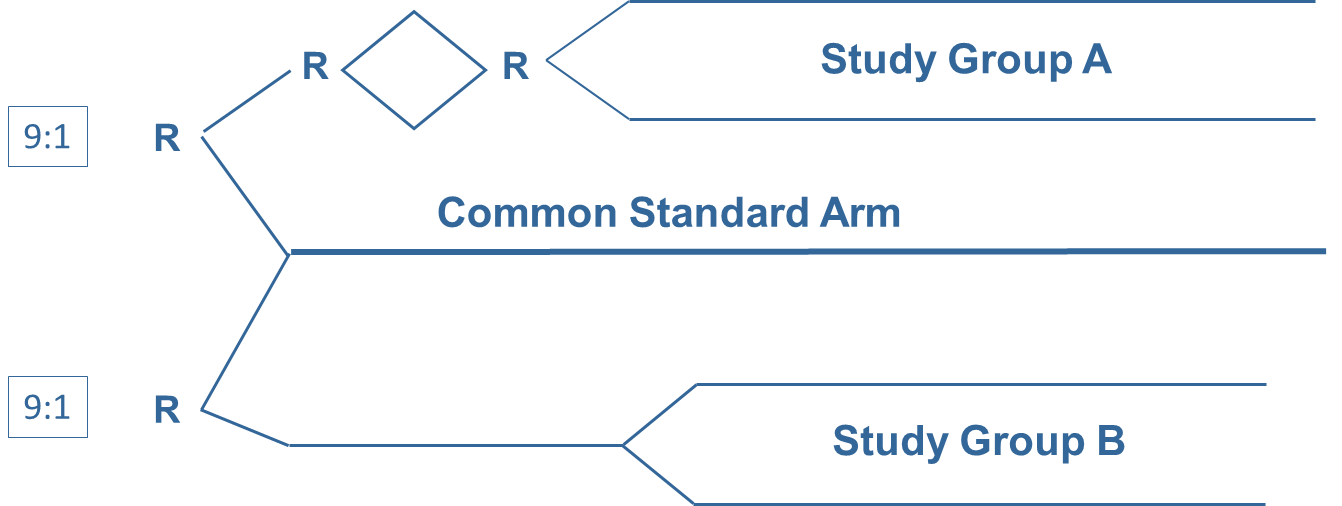
**suppl. Figure 1: Design of the study including common standard arm, study group A and study group B and treatment characteristics**

**Common standard arm (CSA):** 1-2 x araC 100 mg/m2/d C.I. d 1-7 + daunorubicin (dauno) 60 mg/m2/d IV d 3-5; 2 consolidations araC 1 g/m2/d BID IV d 1, 3 and 5 (Mayer RJ et al, NEJM 1994).

**Group A:** TAD (HAM) – HAM vs. HAM (HAM) - HAM ± G-CSF. TAD were given as consolidation followed by maintenance AD-AT-AC-AT over 3 a.

**Group B:** 1-(2) x araC 1  g/m²/d BID IV d 1 + 3 + 5 + 7 and mitoxantrone (mito) 10 mg/m2/d IV  d 1 – 3; 2 x ara-C 500 mg/m² BID 1h IV d 1 + 3 + 5 in combination with mito 10 mg/m2/d IV d 1 + 2 Pegfilgrastim 6 mg s.c. on day 10 of induction and on d 8 of consolidation.

**Abbreviations:** TAD (araC 100 mg/m2/d C.I. d1-2, BID d 3-8 + daunorubicin 60 mg/m2/d IV d 3-5 + 6-thioguanine 100 mg/m2/d po BID d 3-9); HAM (araC 1 g/m2/d IV BID d 1-3 and mitoxantrone 10 mg/m2/d IV d 3-5)


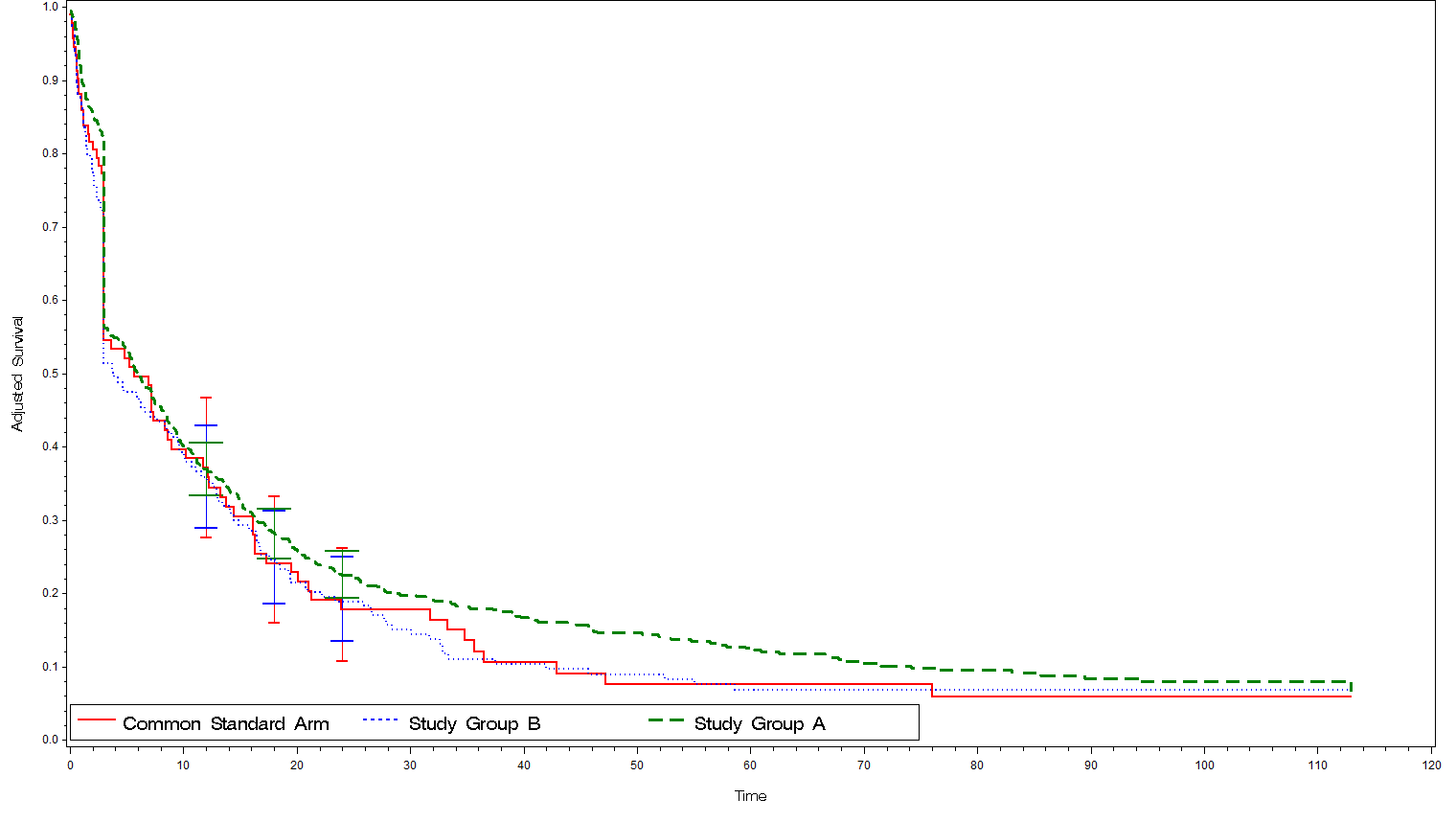
suppl. Figure S2: EFS adjusted

Months from Treatment

suppl. Figure S3: OS adjusted


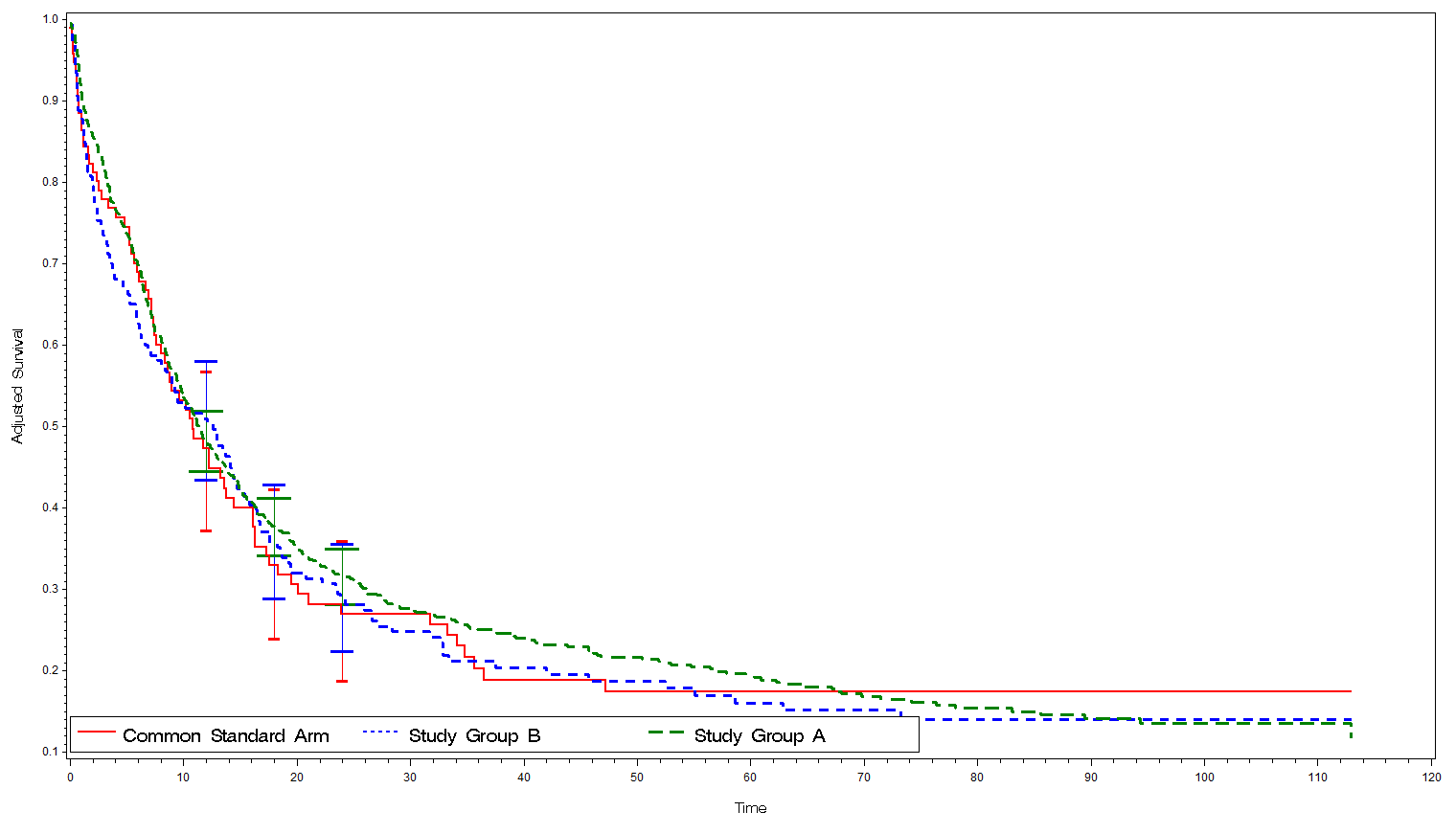


Months from Treatment

suppl. Figure S4: RFS adjusted


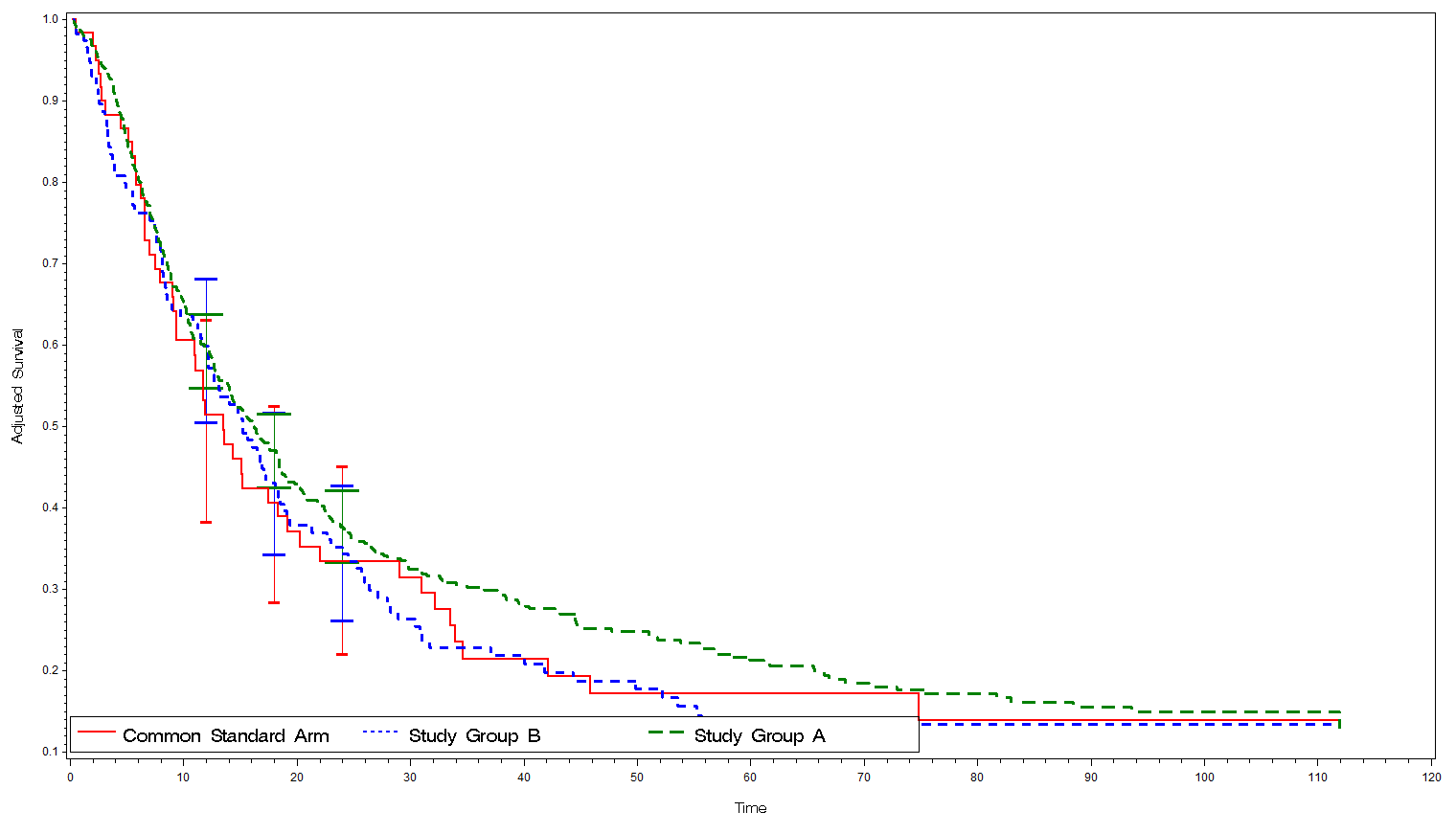


Months from CR/CRi

Months from CR/CRi
